# Be aware of wires in the veins: a case of superior vena cava syndrome in a patient with permanent pacemaker

**DOI:** 10.3402/jchimp.v2i3.19159

**Published:** 2012-10-15

**Authors:** Agegnehu T. Gebreyes, Hom Nath Pant, Donna M. Williams, Sapna P. Kuehl

**Affiliations:** Department of Medicine, Saint Agnes Hospital, Baltimore, MD, USA

**Keywords:** pacemakers, veins, vena cava syndrome

## Abstract

Superior vena cava (SVC) syndrome is an unusual complication of pacemaker and implantable cardioverter–defibrillator implantation. It is believed to be due to SVC thrombosis with or without stenosis induced by endothelial disruption from repeated mechanical trauma by the leads. A 58-year-old man presented with gradual swelling of his face, neck, and upper extremities of 10 days duration. A pacemaker had been implanted for symptomatic bradycardia over 5 years ago. Venous Doppler and venogram revealed thrombosis and stenosis of the SVC. He was treated with multimodal therapy and was discharged with complete resolution of his symptoms.

## Introduction

Superior vena cava (SVC) syndrome was first described by William Hunter in 1757 in a patient with a syphilitic aortic aneurysm (1). Malignant diseases such as bronchial carcinoma and lymphomas are the most common etiology accounting for 95% of cases (2). However, intravascular devices were the most common etiology in benign cases (77%) in a report published by Rice et al. (3).

Asymptomatic venous thrombosis is a more common finding in patients with a pacemaker (4). SVC syndrome in a patient with a permanent cardiac pacemaker was first described by Wertheimer et al. (5). The reported incidence of symptomatic SVC obstruction from pacemaker/ICD insertion widely varies in the literature ranging from 1 in 3100 to 1 in 650 (6–8).

In 2009, more than 350,000 new pacemakers/ICDs were implanted in the United States (9). As the indications for these devices are increasing, pacemaker/ICD lead-induced SVC stenosis and thrombosis are becoming a more frequent benign cause of SVC syndrome. We report a case of SVC syndrome with SVC stenosis and thrombosis secondary to remote pacemaker implantation successfully treated with Angio-Jet^®^ thrombectomy, balloon angioplasty, thrombolytics, and anticoagulation.

## Case presentation

A 58-year-old man presented with a gradual onset of swelling of his face, neck, and upper extremities over a period of 10 days. He had been admitted for a similar complaint to another institution for 6 days and was discharged after diagnosis of angioedema secondary to lisinopril. His workup had included a chest X-ray, a CT of the chest, and a venous Doppler of the neck and upper extremities that were reportedly negative. The patient was treated with steroids and discharged after discontinuation of lisinopril. Two days after discharge, he noticed worsening of facial swelling, including his tongue and lips associated with shortness of breath and difficulty swallowing. He previously had a pacemaker inserted in 2005 for symptomatic bradycardia in another hospital.

**Fig. 1:**
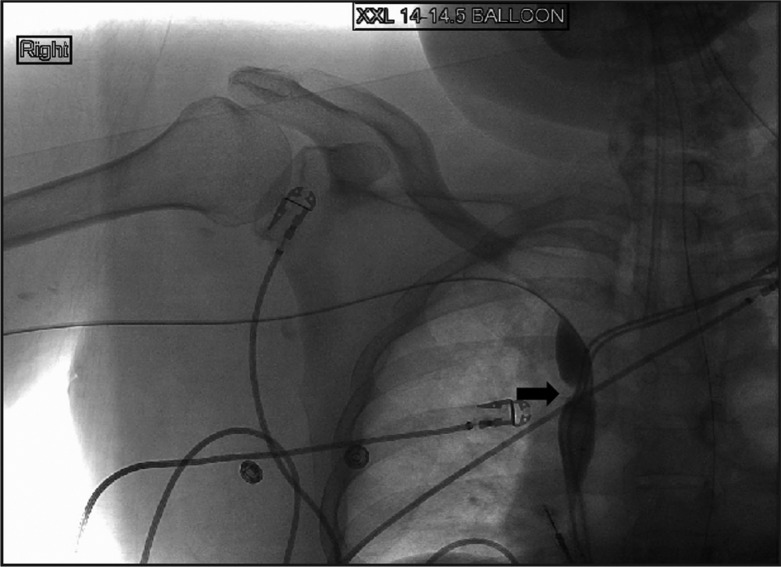
Superior venogram showing tight stenosis of SVC (arrow).

**Fig. 2:**
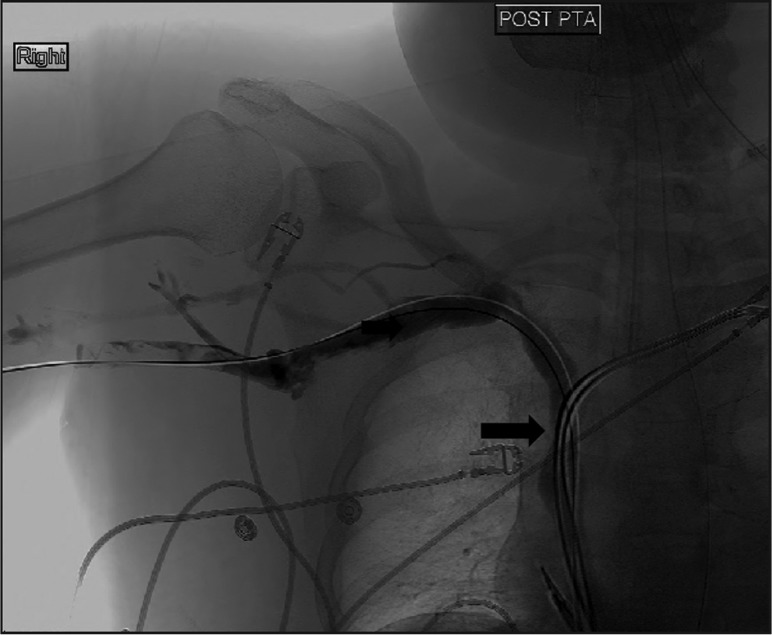
Post angioplasty venogram showing resolution of SVC stenosis (arrow).

Clinical examination revealed a blood pressure of 148/100 mmHg, a regular pulse of 104 beats/min, a respiratory rate of 22 breaths/min, and oxygen saturation of 98% on 2 litres of oxygen. Other significant findings were diffuse facial, neck, and upper extremity swelling. He was intubated. Computed tomography of the neck and chest showed no external compression of SVC by a mass lesion. Venous Doppler of the upper extremities showed deep vein thrombosis of bilateral internal jugular, right axillary and right subclavian veins. A diagnostic upper extremity venogram and a SVC confirmed tight stenosis of the SVC at the site of the cava being traversed by the electrodes (Fig. 1). Angio-Jet rheolytic mechanical thrombectomy was performed by right basilic vein approach using a 6-French Angio-Jet catheter. Catheter-directed thrombolytic therapy was given overnight into the right axillary, right subclavian, and innominate veins using a 45-cm-long infusion catheter. The next day percutaneous transluminal angioplasty of the high-grade SVC stenosis was performed successfully using a 14×45 mm balloon. Immediately post-completion, venography showed excellent flow in the SVC (Fig. 2). The patient made an uneventful recovery with regression of his swelling and was discharged with anticoagulation. There was no recurrence seen at the patient's 2-year follow-up.

## Discussion

SVC occlusion as a result of thrombosis or fibrosis is a rare but potentially serious complication of pacemaker implantation. Several small retrospective studies reported the incidence of SVC obstruction after pacemaker implantation to be 23–50% (6–8). However, recent prospective studies showed that the incidence of venous obstruction after implantation of pacing leads is 15–32% (11, 12). SVC obstruction involves thrombosis, stenosis, or a combination of both. SVC stenosis is believed to be due to endothelial disruption caused by repeated trauma from the leads resulting in inflammation, fibrin deposition, and scarring. Recanalization of thrombus is also assumed to cause fibrotic narrowing of the SVC. The site of SVC stenosis is commonly reported to be close to the right atrium. The patient we describe presented 5 years after pacemaker implantation. However, symptomatic SVC syndrome may occur any time as early as 2 days or as late as 206 months (17 years) (7, 13, 14).

Although no clear risk factors are usually identified, temporary pacing before pacemaker implantation, retained functionless lead and lead infection have been identified as predictors of venous occlusion (12, 15). Multiple studies reported lack of clear evidence that the presence of multiple leads increase the risk of SVC obstruction (10–12). Clinical presentation depends on the level of collateralization of blood flow. Facial, neck, and upper extremity swellings and visible dilated superficial veins on the chest are common presentations observed. In our case, the absence of dilated superficial veins was suggestive of the acute development of SVC thrombosis.

Contrast venography is the gold standard for diagnosis of venous obstruction and provides accurate localization of stenosis and is required for planning vascular intervention and to assess response to treatment. Spiral CT venography is capable of detecting central vein thrombosis and chest CT helps to rule out other etiologies. Although MRA gives more detailed information about collaterals and anatomy, its use is limited due to the presence of pacemakers/ICDs.

Treatment of SVC thrombosis or stenosis is individualized and there are no established guidelines at the present time. The preferred modalities of therapy employed have varied in the past 40 years as reported by Riley et al. (15). In their analysis of 74 publications involving different treatment modalities of 104 cases of symptomatic SVC syndrome, they report that the tide has shifted from anticoagulation or thrombolytic therapy alone in the 1970s to percutaneous stenting in combination with angioplasty and or thrombolytic in the early 2000s (14). Our patient was treated with multimodality therapy involving catheter directed thrombectomy and thrombolysis with percutaneous angioplasty and short course of anticoagulation. The patient has been free of symptoms for more than 2 years.

## Conclusion

SVC obstruction, although rare, is a well-documented complication of pacing leads. Early identification and treatment of this condition will prevent significant morbidity and mortality. With a rapidly growing elderly population, more people with permanent pacemakers/ICDs are likely to be encountered in our practice. Physicians should consider SVC obstruction in patients with implanted pacemakers/ICDs presenting with SVC syndrome even 5 or more years after insertion.
